# Oxytocin administration to clinicians in acute psychiatric care settings: a feasibility study

**DOI:** 10.3389/fpsyt.2025.1671944

**Published:** 2025-10-01

**Authors:** Ayelet Nir, Omer Sedoff, Gal Yenon, Efrat Hirsch Klein, Yaen Schreibman, Khalil Qashu, Hagai Maoz, Yuval Bloch, Dana Tzur Bitan

**Affiliations:** ^1^ Department of Community Mental Health, University of Haifa, Haifa, Israel; ^2^ Tardion Clinic, Clalit Health Services, Tardion, Israel; ^3^ Shalvata Mental Health Center, Hod-Hasharon, Israel; ^4^ Gray Faculty of Medical and Health Sciences, Tel Aviv University, Tel Aviv, Israel

**Keywords:** oxytocin, empathy, acute care, emergency psychiatry, session evaluation

## Abstract

**Introduction:**

Oxytocin (OT) has been previously found to facilitate therapeutic outcomes when administered to patients. Recent evidence suggests that therapist’s OT levels similarly influence clinicians’ ability to respond to patients in an empathic and responsive manner. However, no study previously assessed the impact of OT administration to clinicians treating patients in acute settings. This preliminary feasibility study investigated the applicability and trends of effects of OT administration to clinicians performing triage assessment in a public psychiatric emergency room, while focusing on perceived empathy and quality of the therapeutic encounter.

**Methods:**

Three clinicians were double-blindingly administered with intranasal OT at one day, and saline placebo (PLC) at a different day. The patients they met (N = 16) provided self-reports on the clinician’s empathy (BLRI), the quality of the session (SEQ), and their level of psychological distress (HSCL-11). The Wilcoxon signed-rank test was used to compare ratings across the two conditions.

**Results:**

Patients reported significantly deeper and more valuable meeting with the clinicians following OT administration to clinicians (S = 32.0, p = 0.0398) as well as significantly lower levels of distress (S = 70.5, p = 0.0343). Clinicians perceived empathy was higher after OT administration compared to PLC, however, this difference did not reach statistical significance (S= 38, p = 0.1675).

**Conclusion:**

Although these results should be interpreted with caution due to the preliminary nature of the study, they highlight the potential contribution of clinician’s OT in facilitation the therapeutic process in acute settings, and call for further investigation in larger, controlled trials.

## Introduction

Oxytocin (OT) is a nine-amino neuropeptide known to play a central role in social behavior and interpersonal communication (1). One of the most basic functions of OT is the physiological and emotional regulation of attachment processes occurring within the parent-infant bond (2). Previous studies have documented that OT has activating effects on the caregiving system and is specifically involved in responsiveness to baby’s signals of distress (3), the enhancement of parental proximity (4), and the facilitation of empathy and associated caregiving behavior (5, 6). These central roles have set the stage to consider the role of OT in the patient-therapist dyad, and specifically its effects on therapists’ responsiveness to patients’ distress (7).

Studies assessing the role of OT during therapeutic processes provide some evidence for its involvement among both patients and therapists, as well as its impact on therapy outcomes. Böge, et al. (8) measured OT levels among patients with schizophrenia at baseline and four weeks after mindfulness-based group therapy (MBGT) combined with treatment as usual, and found that OT levels were significantly increased at post-intervention, thus pointing to a potential association between this intervention and changes in the oxytocinergic system. Furthermore, Zierhut et al. (9) conducted a randomized controlled trial to assess the impact of OT administration to patients with schizophrenia undergoing MBGT, and found an improvement in negative affect and stress in the OT group compared to placebo. Atzil-Slonim et al. (10) assessed the OT levels of patients with depression undergoing psychotherapy and found that higher patient’s OT reactivity to the therapeutic encounter predicted larger improvement in depressive symptoms. Similarly, Zilcha-Mano et al. (11) assessed OT secretion of 22 patients with major depressive disorder undergoing psychotherapy, and found that greater increases in OT throughout treatment were associated with more patient-therapist conflicts and more efforts made by therapists for resolving them (12). These findings were also supported by experimental studies assessing the role of OT administration on psychotherapy outcomes; In the largest randomized controlled trial investigating OT administration effects to date, Grossman-Giron et al. (13) found that OT administration to patients as add-on to psychotherapy significantly improved therapeutic outcomes compared to placebo.

Although OT is strongly associated with empathy - a function traditionally endorsed by the therapist rather than the patient - not much is known about its effect on therapists. Nonetheless, physicians’ empathy has been previously associated with treatment outcome, as measured by patient-reported improvement in symptoms (14), treatment compliance, and overall satisfaction with treatment (15). One recent study investigated modulations in OT among therapists (16), and found that patients’ negative emotions at post-session were associated with increase in therapists’ OT throughout the session. Furthermore, they reported that this increase predicted subsequent improvements in depression severity. These findings suggest that increases in OT levels in response to patients’ distress can act as mechanism of change in producing better therapeutic outcome. Nonetheless, to date, no study has experimentally examined whether increasing OT levels among clinicians and therapists would modulate potential therapeutic mechanisms of change such as empathy and session quality, nor their effects on patients’ symptomatic improvement.

This proof-of-concept feasibility study aims to bridge this gap in scientific knowledge, by exploring the effects of OT administrations to clinicians working in emergency settings on patients’ perceived empathy and overall experience with the therapeutic encounter. As no study previously administered OT to therapists prior to a therapeutic engagement, the reported study is aimed to present the feasibility and potential scientific utility of such exploration. Based on the reported literature, we hypothesized that patients treated by therapists who were administered with OT will (a) perceive them as more empathic, (b) will generally experience the therapeutic encounter as more positive, and (c) will report lower levels of distress after the meeting, compared to patients treated by therapists administered with placebo.

## Methods

### Participants

The study included the recruitment of clinicians from the emergency room of Shalvata Mental Health Center in Israel. Inclusion Criteria for clinicians included any therapeutic profession (psychiatrists, psychologists, social workers and nurses) and agreement to receive intranasal OT (IN-OT). Exclusion criteria for clinicians included self-reported pregnancy or breastfeeding; or being the senior psychiatrist in the ER (which was not possible due to medico-legal restrictions). Pregnancy testing was not conducted due to confidentiality and feasibility concerns; thus, exclusion was determined by clinician’s self-report, in accordance with the IRB ethical approval. Inclusion criteria for patients was ability to complete measures, and exclusion was evidence of a current psychotic episode, patients arriving with compulsory orders, or any potential immediate risk of any kind, as determined by a senior psychiatrist or the triage nurses.

Ten clinicians were initially approached for eligibility of the OT administration, of whom five refused to be enrolled due to concerns about intranasal method of administration. Five clinicians signed informed consent, this included three nurses and two psychiatry students. Of these five clinicians, three fully received the experimental substance, one received the substance but saw no patients in her shift, and one received only one arm and was therefore excluded from analysis. This resulted in a total of three clinicians with analyzable data. These clinicians were psychiatric nurses performing psychiatric evaluations in the context of an emergency psychiatry ER. Overall, 25 patients were examined for eligibility to participate in the study. Of these patients, seven were not enrolled due to age restrictions (younger than 18), leaving a total of 18 patients who agreed to participate and signed informed consent. Of them, one patient was excluded due to concerns about privacy, one was excluded because the clinician participated in one arm, thus leading to a final sample of 16 patients treated by three clinicians.

### Measures

#### The Barrett-Lennard relationship inventory – empathic understanding subscale

A measure aimed at assessing the four essential facilitative conditions for positive therapeutic change (18): level of regard, empathic understanding, congruence, and unconditionality of regard (17). The scale is composed of 64-items which measure these four subscales. For the purposes of the current study, we used the empathic understanding subscale which contains 16 items. The measure was found to be highly reliable, with internal consistency of 0.91 for the entire questionnaire, and 0.83 for the empathy subscale (19). The Cronbach’s alpha in the current sample was 0.77.

#### The session evaluation questionnaire

The SEQ is a 20-item measure of the sessions’ qualities, such as session’s depth and flow (20). The measure includes 20 bipolar adjectives scaled on a Likert scale of 1 (such as “happy”) to 7 (“sad”), while inducing responses regarding the meeting (i.e., “This meeting was …”) and regarding patients’ current feelings (i.e., “I now feel…”). To each of these categories there are four subscales which include depth, flow, arousal, and positivity. The depth subscale measures the value and efficacy of the encounter, the flow subscale measures the perception of relaxation and calmness, the arousal subscale measures how arousing and exciting the encounter was, and the positivity subscale relates to the positive affect during and after the encounter. The measure was found reliable in previous studies (Cronbach’s α between α = 0.90 and α = 0.93; 20, 21). Among the subscales, reliability was sufficient given the small number of items in each subscale depth: α=0.80; flow: α= 0.80; arousal: α= 0.62, and positivity: α=0.76 (21). In the current sample Cronbach’s alphas was 0.75 for the total score, 0.70 for depth, 0.57 for flow, 0.52 for arousal, and 0.70 for positivity.

#### The Hopkins symptoms checklist –short form

The HSCL-11 is a brief version of the SCL-90-R (23) and includes 11 items assessing general symptomatic distress through self-report (22). The HSCL-11 was previously found suitable for detecting weekly changes in symptom severity during the course of treatment (24). The scale is highly correlated with the Global Severity Index (r = .91; 22) and has high internal consistency (α = .92; 25). The measure was completed by patients after the meeting with the clinician. Cronbach’s α for the current sample was α = 0.73.

### Procedure

The study was approved by the Institutional Review Board (IRB) of Shavlata Mental Health Center (approval reference: 0002-22-SHA). Clinicians participating in the study were randomly allocated to receive either IN-OT or placebo (PLC) on two different days. After administration, all the patients evaluated by the clinician participating in the study were requested to complete measures of empathy, session quality, and distress. Staff and patients reported their demographic characteristics. Intranasal OT was administered by the nurse attending the shift during research procedures. Therapists received OT and Placebo in a random order which was blinded for the receiver. Both substances were administered intranasally and therefore were not tasted. The nasal spray of the OT included 24 IU OT, alcohol, benzyl, sorbitol glycerol and distilled water. Placebo was delivered via same syringe type with nasal adapter allowing intranasal self-inhalation and contained a 10cc ampule containing saline (sodium chloride 0.9%).

### Statistical strategy

Descriptive statistics (means, standard deviations, ranges, and frequencies) were calculated for clinician and patient demographic and clinical characteristics. Non-parametric tests were used to assess differences between the OT and PLC conditions in the measured instruments, due to the small sample size and non-normal distribution of data. Comparisons of perceived empathy ratings, session evaluation (SEQ and subscales), and psychological distress (HSCL-11) across the two conditions were tested using the Wilcoxon signed-rank test. Statistical significance was set at *p* < 0.05. Missing data were handled using listwise deletion. All statistical analyses were done using SPSS version 25.

## Results

### Sample characteristics

**Table 1** presents the characteristics of the patients and clinicians participating in the study. All three clinicians were nurses, men, aged 35–48 (M = 40.3, SD = 5.56), working 3–27 years in their profession (M = 11.33 years, SD = 11.08). The number of recruited patients per clinician ranged from two to six (M = 1.93, SD = 1.32). Patient characteristics are elaborated in **Table 1**. The mean age was 38 (SD = 19.66), with ages ranging from 18 to 67. The sample included seven men (44%) and nine women (56%). Most were single (N = 10; 62.5%), while one was married (6.25%), four were divorced (25%), and one was widowed (6.25%).

**Table 1:** Patients and clinicians’ characteristics.

*Clinicians’ characteristics*		N	%
Age	(M, SD)	40.3	5.56
Gender	Male	3	100
	Female	0	0
Profession	Nurses	3	100
No. of years working in profession	(M, SD)	11.33	11.08
*Patients’ characteristics*			
Age	(M, SD)	38	19.66
Gender	Male	7	44
	Female	9	56
Marital Status	Single	10	62.5
	Married	1	6.25
	Divorced	4	25
	Widowed	1	6.25
Number of prior medications	(M, SD)	3.56	1.68
Prior psychiatric ER visits	(M, SD)	5	9.57
Prior psychiatric hospitalizations	(M, SD)	6.62	7.27
Diagnosis, present ER visit	Affective	5	31.3
	Personality disorders	5	31.3
	Psychotic	3	18.75
	Anxiety	1	6.25
	No diagnosis	2	12.5
Psychiatric Comorbidity	Personality disorders OCD	3 2	18.75 12.5
	Addictions PTSD	5 1	31.3 6.25
	None	5	31.3

Of the patients completing the study, five (31.3%) were diagnosed with affective disorders, including major depression and bipolar disorder; one (6.25%) with acute anxiety disorder; three (50%) with psychotic disorders, including schizophrenia, schizoaffective disorder, and acute psychotic episode; and five (31.3%) with personality disorders, four of them with borderline personality disorder and one mixed personality disorder; the remaining two did not receive a present ER diagnosis (12.5%). Comorbid conditions were observed among eleven patients, three with borderline personality disorder comorbidity (18.75%), five with comorbid addictions (31.3%), two with obsessive compulsive disorder comorbidity (12.5%), and one with comorbid post-traumatic stress disorder (6.25%). The mean number of prior visits in the psychiatric ER was 5 (SD = 9.57), and the mean number of previous psychiatric hospitalization was 6.62 (SD = 7.27).

**Figure 1:**
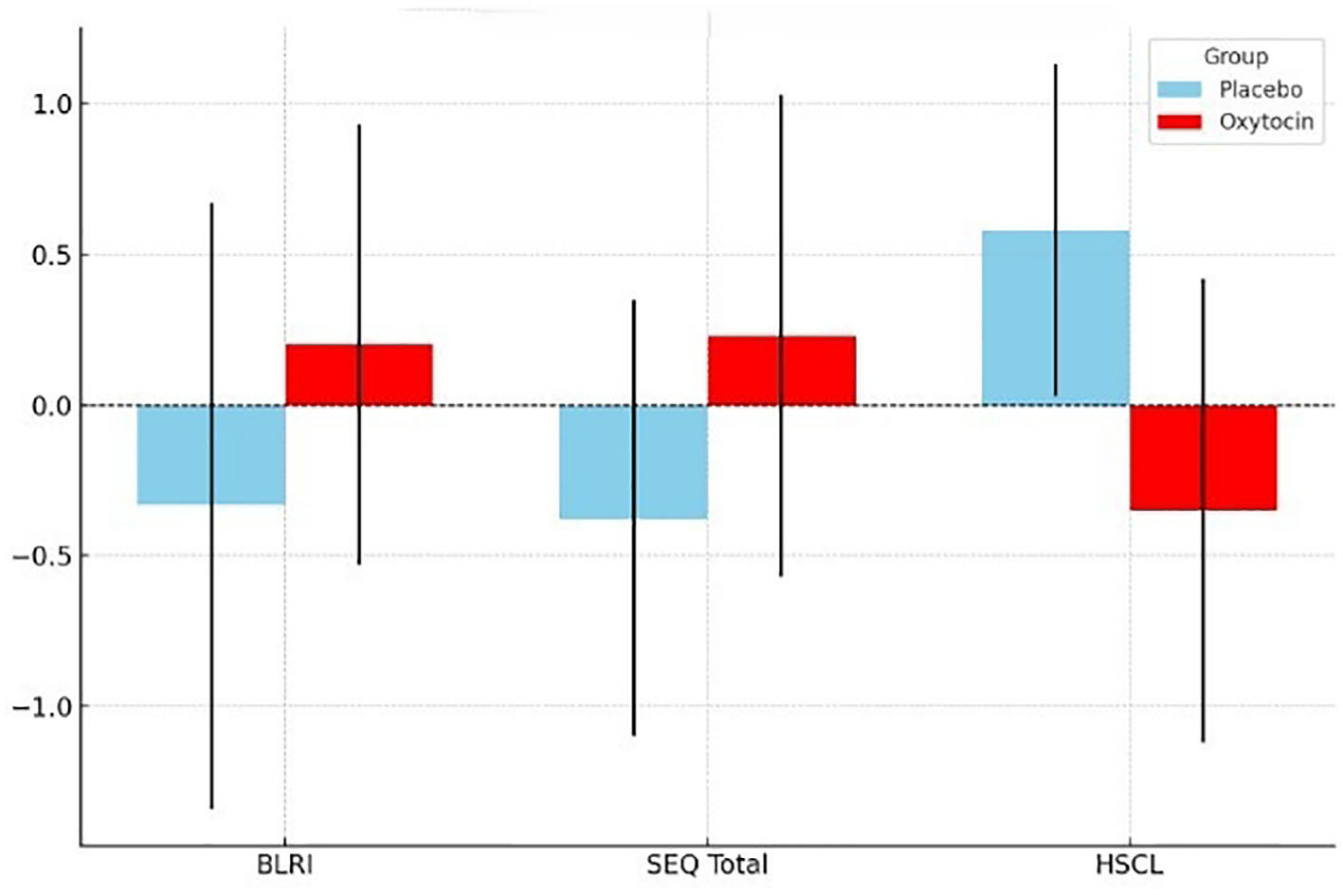
Visual representation of the effect of OT administration to clinicians on the perceived empathy (BLRI), session evaluation (SEQ) and symptoms (HSCL-11). Oxytocin condition is outlined in red and the PLC condition in light blue. BLRI, The Barrett-Lennard Relationship Inventory – Empathic Understanding Subscale; SEQ, Session Evaluation Questionnaire; HSCL, The Hopkins Symptoms Checklist –short form.

**Table 2** displays the means and standard deviations for the main measurements of the study. The mean score for perceived empathy of the clinician was 76.70 (SD = 10.26) following administration of OT, and 71.33 (SD = 9.65) after administration of PLC. These differences did not reach statistical significance (S = 38, *p* = 0.1675). The mean score for the session evaluation (SEQ total score) was 4.51 (SD = 0.86) for the OT condition, and 4.04 (SD = 0.53) for the PLC condition, again not reaching statistical significance (S = 37.0, *p* = 0.1456). The SEQ subscale of depth indicated significantly higher ratings in the OT condition (M = 4.90, SD = 1.45) compared to the PLC (M = 3.77, SD = 0.69) (S = 32.0, *p* = 0.0398). All other measures showed similarly higher scores in the OT condition (flow: M = 5.62, SD = 0.98; arousal: M = 3.78, SD = 1.31; positivity: M = 3.72, SD = 1.47) compared to the PLC (flow: M = 5.23, SD = 1.09; arousal: M = 3.80, SD = 0.52; positivity: M = 3.37, SD = 0.97), however, none of them reached statistical significance (flow: S = 43.5, *p* = 0.4404; arousal: S = 55.0, *p* = 0.6906; positivity: S = 45.0, *p* = 0.5418). The symptom severity scale (HSCL-11) showed statistically significant lower ratings of distress in the OT condition (M = 35.80, SD = 5.67) compared to PLC (M = 40.67, SD = 2.73, S = 70.5, *p* = 0.0343). These effects are illustrated in **Figure 1**.

**Table 2:** Means, SD’s and significance of difference in perceived empathy, general evaluation of the session, and symptoms among patients of clinicians receiving OT versus PLC.

	OT (N = 10)	Placebo (N = 6)	Wilcoxon Two-Sample Test (two-sided)	
	M (SD)	M (SD)	Test Statistic (S)	P-value
Perceived Empathy BLRI	76.70 (10.26)	71.33 (9.65)	38	0.1675
SEQ Total	4.51 (0.86)	4.04 (0.53)	37.0	0.1456
SEQ Depth	**4.90 (1.45)**	**3.77 (0.69)**	**32.0**	**0.0398**
SEQ Flow	5.62 (0.98)	5.23 (1.09)	43.5	0.4404
SEQ Arousal	3.78 (1.31)	3.80 (0.52)	55.0	0.6906
SEQ Positivity	3.72 (1.47)	3.37 (0.97)	45.0	0.5418
HSCL-11	**35.80 (5.67)**	**40.67 (2.73)**	**70.5**	**0.0343**

Bold represent significant values.

## Discussion

This study aimed to assess the feasibility and preliminary trends of the effects of administrating OT to clinicians, and specifically to triage nurses performing clinical evaluations in acute settings. To the best of our knowledge, this is the first study aiming to delineate the effects of OT augmentation to clinicians. The feasibility testing of this study indicated that recruiting clinicians to participate in an OT administration trial is complex. Nonetheless, and although the results should be interpreted with caution due to the small sample size, the results also indicated that OT administration may be associated with increase in perceived empathy, as well by with significant improvement in session quality and level of distress.

Approximately 50% of the clinicians who were invited to participate in the study agreed to receive OT as part of a clinical trial, while half of them refused to participate due to concerns about intranasal method of administration. This low recruitment rate aligns with existing literature highlighting the challenges of enrolling clinicians in clinical trials, especially those involving substance administration. For example, in a randomized controlled trial assessing the effects of Modafinil on cognitive performance in sleep-deprived doctors, Sugden et al. (26) approached 55 physicians, of whom nine refused to participate. Similarly, Franke et al. (27) distributed over 3,000 questionnaires at international surgery conferences, yet only 36.4% of surgeons completed the survey despite guaranteed anonymity. In a more recent trial, Back et al. (28) screened 2,247 clinicians for participation in a study administering Psilocybin for depression and burnout, yet only 30 were eventually enrolled. These findings illustrating that even high initial interest often fails to translate into participation when the intervention involves psychoactive compounds. These findings might suggest that shifting from a provider position to a research participant might be uncomfortable to clinicians, especially when substance administration is involved. Furthermore, as this study required evaluation of clinicians therapeutic practice by their patients, it is also possible that the study invoked fears of being judged or criticized (29).

Although underpowered, the results demonstrate that patients reported higher perceived empathy when clinicians were under the OT condition. A recent review of 44 studies focusing on the relationship between OT and empathy found that an increase in OT release over time was associated with greater empathy (30). In the settings of parent-infant bond, studies demonstrate that increased parents’ OT levels are significantly related to more affectionate behaviors and greater responsiveness to their infant (31), as well as to infants’ development of secure attachment (32). These findings imply that OT release in caregiving contexts may reflect both emotional attunement and motivational engagement with the distressed other - mechanisms that may also be relevant to the therapeutic relationship.

Patients reported significantly higher levels of in-session depth in the OT condition compared with the control. The SEQ depth subscale has been linked with clients perception of the session as powerful and valuable versus weak and worthless (33).Thus, it is possible that therapists’ empathy and engagement in the clinical meeting led to more valuable and powerful meeting. In a case study conducted by Grossman-Giron et al. (34), the medical notes of therapists of patients who received either OT or PLC were examined. The findings indicated that the therapist of the patient receiving OT report that “the patient was very communicative and pleasant”; “the patient was more expressive, and his affect display was broader than usual”; “he seemed more present and engaged in what was said in the session.” Although these findings relate to patients’ rather than therapists’ administration, they may suggest that OT administration to either side of the therapeutic dyad results in a more pleasant and communicative interaction between the clinicians and therapists.

Patients of clinicians receiving OT reported significantly lower levels of symptomatic distress compared to patients of clinicians receiving PLC. These findings resonate with the findings reported by Fisher et al. (16), who found that therapists increased levels of OT were associated with a significant decrease in depressive symptoms among their patients. These findings may suggest that biological augmentation of OT may improve clinicians’ capability to be responsive, attuned and empathic towards their clients, and that these interpersonal gestures lead to better therapeutic outcomes (35–37). This may imply that the main mechanism which enables therapeutic change is the activation of the therapists’ attachment system and ability to respond to the patient in an empathic manner.

The results of this study can be considered in light of their empirical and clinical relevance. Although this study primarily aimed to explore the contribution of OT when manipulated at the therapist’s (rather than the patient’s) level, additional theoretical and empirical research is needed to explore the feasibility and utility of such administration in routine clinical settings. Nonetheless, the results can inform future studies focusing on the biological and hormonal determinants of effective therapists (38), to further advance current knowledge on how to improve psychotherapeutic interventions. According to the social salience hypothesis of Shamay-Tsoory and Abu-Akel (39), OT activation is context-dependent, and exerts its effects through the modulation of attention to external contextual social cues. Such a theory potentially implies that OT administered in emergency settings might increase the overall salience of stressful cues or alternatively increase the salience of interpersonal ones. Additional studies are needed to fully delineate the impact of OT facilitation and its context-dependent effects in these settings.

Although the results of this study can inform future research, several limitations should be noted. The small number of participating clinicians and patients, as well as the short timeframe of data collection, limits the generalizability of the results. Additional studies are needed to further validate the results, using larger sample size. Since no checks were performed to make sure that clinicians were unaware of the substance they received, we cannot exclude the possibility that the ratings were biased by self-identification. Future studies should aim to replicate our findings while verifying randomization. A further limitation is the absence of clinician-reported measures, which were not collected due to concerns about its feasibility in emergency settings. Although the final sample of participants in this study included only male clinicians, future studies should also measure estrogen levels and menstrual cycle phase among women, to account for potential hormonal effects. Side effects were not documented in this study, future studies should aim to further test whether OT has side effects when administered to practitioners in emergency settings. Due to the exclusion of two female practitioners due to lack of patients in their shift, the final sample included only male practitioners. Additional studies are needed to examine the effects of OT administration on both female and male practitioners. Practitioners were randomized to either receive OT or PLC in two different shifts, where they met different patients. Thus, the possibility that the reported differences between groups were attributed to the patients’ pre-existing differences cannot be excluded. Notwithstanding these limitations, the results of the study highlight the potential empirical and clinical advancements which can be made by further testing the effects of clinicians OT to routine practice, in order to improve patient care.

## Data Availability

The datasets presented in this article are not readily available because Due to ethical restrictions, raw data cannot be shared. Aggregated data is available from the corresponding author upon reasonable request. Requests to access the datasets should be directed to dana.tzur@gmail.com.
